# Facts, Not Theories

**Published:** 1892-01

**Authors:** I. Dever

**Affiliations:** Clinton, N. Y.; Bureau of Homœopathic Philosophy, I. H. A.


### FACTS, NOT THEORIES.

I. Dever, M. D., Clinton, N. Y.

(Bureau of Homœopathic Philosophy, I. H. A.)

Facts are stubborn things which though for a time may be kept in the background or wholly ignored, they will nevertheless remain unchanged by time. They remain the same stubborn things.

Less than four hundred years have elapsed since Ambrose Paré discovered the fact that boiling oil and the actual cautery were unnecessary remedies in the treatment of gunshot wounds, a practice which existed previous to his advent as a surgeon, and had been regarded as necessary on account of the supposed poisonous nature of the wound.

The same author demonstrated the fact that the ends of bleeding vessels could be ligated and should not be subject to the red-hot iron. Not until 1619 did the medical profession learn the fact, and then slowly, which Harvey taught them in regard to the circulation of the blood.

In 1790, Hahnemann discovered that a drug produces upon a healthy man the very symptoms which it was expected to cure upon the sick, which fact suggested the idea of *Simila similibus curantur*, or Homœopathy, which is a systematic arrangement of facts that can be demonstrated in daily practice, at the bedside of the sick, which fact fully establishes its claim as a science.

The law which governs the science of Homœopathy partakes somewhat of the nature of the Great Law-Giver, for it is a jealous law and demands strict obedience on the part of those who seek its kindly helping hand in restoring the sick to health.

The formula *Similia similibus curantur* is fraught with meaning to the Hahnemannian who never forgets that similars, and only similars should be expected to cure.

Neither does he forget that " the sole duty of the physician is to restore health in a mild, prompt, and durable manner, and that it does not pertain to the office of the physician to invent systems or vainly attempt to account for the morbid phenomena in disease." We are evidently not called on to invent theory which has no part as a therapeutic measure in restoring health to the sick—theory always taking second place to fact—but it is our duty and privilege to study the complex changes which take place in the system necessary to convert abnormal vital action into a state of health.

It is highly necessary that the homœopathist should understand the law of similars and understandingly make the application. It will not do to repeat the formula and then prescribe all manner of medicine in the name of Homœopathy, for the law of similars is a perfect law governing homœopathic therapeutics which admits of no mixtures of medicines.

Neither does it prescribe medicine which has not first been proven on the healthy, that we as physicians may know their sick-producing properties, and when to prescribe them for the cure of the sick.

No medicine is homœopathic (it matters not how high or low the attenuation may be) if it does not present in its disease-producing symptoms a picture which is the counterpart of the abnormal action for which it is prescribed.

This is a rule of action which does not admit of an exception. We are likewise admonished to use only simple single medicines for the cure of the sick. By simple medicine we understand that we are to be governed by the provings, and in no case to resort to two or more remedies, as I have known professed homœopaths to do. One remedy for the headache and one for the sore throat in La Grippe.

Homœopathy teaches the fact that, though the remedy may be selected with reference to its homœopathic indications, much depends on the administration of the same, and it admonishes the practitioner not to administer the remedy too strong if he would not defeat the object for which he is supposed to prescribe. Another fact which presents itself for our consideration in this connection is that the judicious prescriber is slow to repeat a well-selected remedy, fearing that if often repeated he may convert good into evil by so burdening the vital forces that he will not only fail to cure the sickness, but may even place the life of his patient in jeopardy. This is a principle well established in Homœopathy, which is sustained by every member of the homœopathic profession who has placed himself in position to observe this important fact.

Why this is so we will not attempt to explain, as we are here only to deal with fact established on the experience of the multitude of practitioners who are not blind to those silent forces in nature, which, in fact, are not confined to medicine, but are the unseen forces which control the universe.

That the homœopathic remedy well selected in regard to the adaptability of the patient will cause the vital forces to act in opposition to it for days, weeks, and months is a fact which cannot be explained away by any hypothesis which can be invented. Like the minimum dose, whose efficiency is well attested by hundreds who have seen it act, and have put it to the practical test, it stands a fact which cannot be refuted by any number of witnesses who have never seen it, nor ever put it to the practical test for the cure of disease.

Homœopathy rests on the firm foundation of fact, nevertheless it is sustained by theory in explanation of the fact, differing, however, in this respect from those systems of medicine which offer and accept theory regardless of fact. But we are not inclined to talk about our neighbors, and would pass them by unnoticed were it not for their extravagant claims to a science which they never possessed, an example of which may be found in the late lymph theory. To Homœopathy belongs the credit of first preparing the remedy, and to Homœopathy represented by Dr. J. A. Biegler, of Rochester, N. Y., belongs the first reported case which dates back to 1878. (See report in March number *Medical Advance*, page 132, which is an extract from the *Organon*, 1879.) The remarkable fact in reference to this report is, that notwithstanding the remedy was not one in general use, Dr. Biegler, governed by the law of similars, knew just how to make the application of Tuburculinum for the cure of tubercular meningitis.

This case was not phenomenal, but was the result of a close application of the principles to which we as an organization subscribe.

Another fact which presents itself in this case is the care with which the prescription was made by Dr. Biegler, who tells us it was after careful study that he gave a highly attenuated dose of this remedy, which from present indication will soon be abandoned by our step-brothers, who, though they have stumbled on a good remedy are destitute of a law, and do not know how to prescribe it. They have theory, but we have the fact.

